# SignalFormer: Hybrid Transformer for Automatic Drone Identification Based on Drone RF Signals

**DOI:** 10.3390/s23229098

**Published:** 2023-11-10

**Authors:** Xiang Yan, Bing Han, Zhigang Su, Jingtang Hao

**Affiliations:** Sino-European Institute of Aviation Engineering, Civil Aviation University of China, Tianjin 300300, China; 166041238@cauc.edu.cn (X.Y.); zgsu@cauc.edu.cn (Z.S.); jthao@cauc.edu.cn (J.H.)

**Keywords:** internet of drones, automatic drone identification, time–frequency analysis, deep learning

## Abstract

With the growing integration of drones into various civilian applications, the demand for effective automatic drone identification (ADI) technology has become essential to monitor malicious drone flights and mitigate potential threats. While numerous convolutional neural network (CNN)-based methods have been proposed for ADI tasks, the inherent local connectivity of the convolution operator in CNN models severely constrains RF signal identification performance. In this paper, we propose an innovative hybrid transformer model featuring a CNN-based tokenization method that is capable of generating T-F tokens enriched with significant local context information, and complemented by an efficient gated self-attention mechanism to capture global time/frequency correlations among these T-F tokens. Furthermore, we underscore the substantial impact of incorporating phase information into the input of the SignalFormer model. We evaluated the proposed method on two public datasets under Gaussian white noise and co-frequency signal interference conditions, The SignalFormer model achieved impressive identification accuracy of 97.57% and 98.03% for coarse-grained identification tasks, and 97.48% and 98.16% for fine-grained identification tasks. Furthermore, we introduced a class-incremental learning evaluation to demonstrate SignalFormer’s competence in handling previously unseen categories of drone signals. The above results collectively demonstrate that the proposed method is a promising solution for supporting the ADI task in reliable ways.

## 1. Introduction

With the rapid explosive growth of drone applications in various civilian fields, it is anticipated that millions of drones will access low-altitude airspace, executing diverse civil services within the coming decade [1,2]. However, the surge in drone flights has raised concerns regarding the adequacy of existing air traffic management technologies to ensure the safety and security of low-altitude airspace [3].

To address this challenge, the concept of the Internet of Drones (IoD) has emerged, aimed at enhancing communication, navigation, and surveillance capabilities while concurrently integrating drone flight management [4,5,6]. Automatic drone identification (ADI) [7] is the essential component of the IoD framework to ascertain the presence of drones. ADI technology can be divided into two main categories: active and non-cooperative. Active ADI technology primarily involves detecting drone targets through active radar echoes [8,9]. In contrast, non-cooperative ADI technology passively detects drone targets based on physical mediums such as acoustic signals [7,10], optical signals [11,12], and radio frequency (RF) signals emitted by drones [13,14,15]. Compared to other technologies, non-cooperative ADI technology based on drone RF signals offers a wider surveillance range and higher identification accuracy.

The problem of drone RF signal identification has typically been formulated as a classification problem in the fields of machine learning (ML) and deep learning (DL), which involves using ML or DL models to identify the presence of drone RF signals in the spatial electromagnetic spectrum, thereby determining the existence of drone activity in the airspace. Therefore, from the perspective of the adopted models, previous research can be divided into two main types: ML-based identification models [16,17,18] and DL-based identification models [13,14,19,20,21,22]. ML-based identification models mostly design the handcraft features from the one-dimensional (1D) time-domain waveform of drone RF signals by statistical knowledge, then use ML techniques, such as support vector machine (SVM) or multi-layer perception (MLP), to perform the final classification task by the handcraft features. On the other hand, DL-based identification models primarily employ a deep neural network (DNN) to automatically extract signal features from the 1D time-domain, 1D frequency-domain, or two-dimensional (2D) time–frequency (T-F) domain; they then use MLP to accomplish the classification task.

With the continuous development of deep learning algorithms in recent years and the improvement in computing hardware, more research has adopted the DL-based identification approach, thereby eliminating the dependence of model performance on the quality of handcraft features. In the early stages, the DL-based identification model also relied on automatically extracting features from the time-domain waveform to accomplish drone RF signal identification [19]. While time-domain methods are proficient in effectively handling waveform identifications, they are susceptible to disruptions caused by noise components. Due to the powerful 2D feature extraction capabilities of modern DNNs, some studies suggest first using T-F analysis algorithms to transform signals from the time domain to the T-F domain, then extracting features from both time and frequency dimensions [23,24]. This approach prevents the identification model from being affected by noise outside the signal’s frequency band, thereby enhancing the model’s noise resistance and elevating better identification accuracy.

However, several challenges remain unresolved in previous research, including model architectural designs and engineering applications. Firstly, traditional drone RF signal identification models predominantly rely on convolutional operators, which are local feature extraction algorithms with limited receptive fields, thereby impacting the model’s identification accuracy. Secondly, previous research usually focused on the magnitude information in the T-F spectrum and neglected the use of phase information. Next, earlier studies mainly considered drone RF signal identification in Gaussian white noise environments. However, co-frequency interference, such as Wi-Fi and Bluetooth, can also introduce significant complexities to drone RF signal identification that cannot be overlooked in practical applications. Lastly, due to the data-driven nature of DL-based drone identification methods, well-trained signal identification models are usually effective in identifying signal categories covered by the dataset. Nevertheless, the identification performance may decrease when faced with drone signal categories not included in the dataset. Therefore, it is essential to assess the class-incremental learning (CIL) capability of drone signal identification models.

In this paper, we aim to address crucial gaps within current drone RF signal identification research. Firstly, we confront the challenge of limited receptive fields in CNN models by incorporating the self-attention (SA) mechanism from the transformer model [25] into drone RF signal identification tasks. In contrast to the local connectivity of convolution operators, the SA mechanism excels at global feature modeling and capturing long-range dependencies in input data. To adapt the signal T-F spectrum to the SA mechanism, we design a CNN-based tokenizer (C-tokenizer) to generate T-F tokens enriched with local context information for each T-F bin within the T-F spectrum. Then, we design an effective time/frequency transformer encoder (T/F-encoder) with the gated self-attention (GSA) mechanism to capture the global time/frequency correlations between the T-F tokens. Combining the C-tokenizer, T/F-encoder, and other components, a hybrid transformer model named SignalFormer is conducted for drone RF signal identification tasks. Secondly, we mitigate the absence of phase information in signal representation by concatenating the real part, imaginary part, and magnitude of the T-F spectrum as the model input, which can substantially enhance the SignalFormer’s ability to handle complex RF signal data. Thirdly, we extend our study to evaluate SignalFormer’s performance under the environment of co-frequency interference, such as Bluetooth and Wi-Fi signals within the same frequency band. This investigation sheds light on the model’s robustness and practical applicability. Lastly, we validate SignalFormer’s adaptability through class-incremental learning (CIL), leveraging a fine-tuning approach. Our findings underscore SignalFormer’s capability to effectively handle previously unseen drone signal classes. To substantiate the efficacy of our proposed approach, we conduct comprehensive experiments on two publicly available datasets. The results consistently demonstrate SignalFormer’s superiority over existing benchmarks in the field of drone RF signal identification.

In summary, the main contributions of this paper are as follows:

- We propose a hybrid transformer model named SignalFormer, which efficiently captures both local context and the global attention map in the T-F spectrum of drone RF signals. Our model is highly effective at identifying drone RF signals in the aerial RF environment.
- We uncover the critical importance of phase information in the T-F spectrum for drone RF signal identification. Incorporating phase information into the model’s input data significantly enhances its identification performance.
- We conduct a series of experiments under co-frequency signal interference conditions to validate SignalFormer’s ability to withstand such interference. These experiments demonstrated the model’s strong robustness and practicality.
- We introduce class-incremental learning evaluation to the field of drone RF signal identification. The evaluation results revealed SignalFormer’s capability to effectively handle previously unseen categories of drone signals.

The rest of this paper is organized as follows: Section 2 reviews the related works in the literature. Section 3 presents the identification framework and Section 4 explains the architecture of the SignalFormer model. Section 5 shows the detailed experimental setting and experimental results. The conclusion is presented in Section 6.

## 2. Related Work

Automatic drone identification through RF signals can be grouped into the following approaches: ML-based identification techniques and DL-based identification techniques. In general, these techniques extract the signal features between a drone and its controller.

### 2.1. ML-Based Drone RF Signal Identification Techniques

Most of the ML-based techniques rely on manually constructing statistical features from the time-domain waveform and utilizing ML algorithms for signal identification. Experimental results indicate that such algorithms typically have lower computational complexity, but the performance of these algorithms is often limited by identification accuracy. However, due to susceptibility to noise influence in the signal waveform, the performance of these algorithms is often limited by identification accuracy.

In [16], 15 types of statistical features, such as mean, standard deviation, and entropy, are chosen as the basis for identification. Subsequently, the neighborhood component analysis (NCA) algorithm is applied to reduce feature dimensionality. The reduced features are employed to train three machine learning algorithms, namely discriminant analysis (DA), support vector machine (SVM), and neural network (NN). The experimental results indicate that the ML algorithms can exhibit 90% high accuracy when the signal-to-noise ratio (SNR) exceeds 10 dB. In [17], the use of fractal dimension (FD), axially integrated bispectra (AIB), and square-integrated bispectra (SIB) as three types of RF fingerprint features is proposed to replace commonly used statistical measures and enhance the applicability and reliability of feature data. With the help of improved RF fingerprint features, the system shows an accuracy of 100% when two types of drone are identified for SNR 0 dB.

To facilitate the identification of mini drones using Wi-Fi as their communication system, the algorithm proposed in [18] involves extracting statistical features such as packet length and inter-arrival time from Wi-Fi traffic. The identification process employs the cross-entropy function as the loss function, and the maximum likelihood estimation method is applied to estimate the parameters of the exponential distribution. This approach achieves effective drone signal detection with accuracy ranging from 87% to 95% at distances of 70 m and 40 m in line-of-sight (LoS) and non-line-of-sight (NLoS) scenarios, respectively.

### 2.2. DL-Based Drone RF Signal Identification Techniques

In recent years, with the increase in data volume and the growing complexity of tasks, the field of drone control signal identification has gradually shifted toward using deep neural networks instead of traditional ML algorithms. In addition to processing signals in the time domain, some recent research work has proposed T-F domain identification methods, which involve transforming time-domain signals into the T-F domain for signal identification.

In [19], an auxiliary classifier Wasserstein generative adversarial network (AC-WGANs) is utilized for recognizing drone temporal waveforms. To mitigate computational complexity, the authors preprocess and dimensionally reduce the received signal waveforms, representing information in a lower-dimensional space. Subsequently, the processed signal data are input into the AC-WGANs model for feature extraction and signal identification. Experimental results indicate that the model achieves approximately 95% identification accuracy for SNR 5 dB. In [20], an end-to-end signal detection and identification model is proposed to save computation time during the feature extraction step. The SqueezeNet model with one-dimensional convolution operators is used to directly extract RF fingerprint features from the time-domain envelope. This approach significantly reduces the model’s computation latency, with an inference time of only 0.37 ms for a single drone RF signal. Within the 0 dB to 30 dB SNR range, the method achieves an average identification accuracy of 97.53%. However, as the SNR drops to 0 dB, the model’s identification performance is notably compromised due to the impact of noise impact.

In [21], the drone signal undergoes the short-time Fourier transform (STFT) algorithm to derive the T-F spectrum. Subsequently, a residual neural network (ResNet) is applied to extract feature information from the T-F spectrum. The authors assessed the algorithm’s performance in drone RF signal detection across various SNRs. The system attains nearly 99% identification accuracy at an SNR of 0 dB. In [22], the authors proposed utilizing wavelet transform analysis for the time–frequency domain transformation of drone signals. They also compared the feature extraction performance of three wavelet transform algorithms: discrete wavelet transform (DWT), continuous wavelet transform (CWT), and wavelet scattering transform (WST). Among them, the identification method based on WST and SqueezeNet demonstrated superior performance, achieving an accuracy of 98.9% at an SNR of 10 dB.

Although prior studies have effectively detected drone control signals within a high SNR range, issues such as low SNR persist. Most existing research results have adopted convolutional-based DL models. Although convolutional operators have strong feature extraction capabilities, simply using convolutional operators will lose global features contained in signal data and limit model performance. However, incorporating self-attention mechanisms into neural network models can overcome this limitation. In addition, there are still some other uncovered issues in previous research works, such as neglecting the phase information in the drone T-F spectrum and resulting in incomplete feature data input, less consideration of signal identification in the co-frequency signal interference environment, and the identification of unknown drone signals, which present the model generalization problem. We start by describing the design of the hybrid transformer model-based drone RF signal identification framework.

## 3. Overview of the Proposed Drone RF Signal Identification Framework

Since the frequency of the drone RF communication link falls within the 2.4 GHz to 2.48 GHz range of the ISM band, it overlaps with a substantial amount of civilian wireless network signals like Bluetooth and Wi-Fi. These RF signals sharing the same frequency band can introduce significant interference to drone RF signals. Consequently, the drone RF signal identification model must initially possess coarse-grained signal identification capability, which involves the ability to differentiate various RF signals on the ISM band and determine whether the received signal originates from a drone. On the other hand, the identification model also needs fine-grained signal identification capability, which involves recognizing the specific drone type. This capability allows for obtaining detailed parameters of the drone, such as its physical attributes and flight speed [10], thus providing essential data support to the IoD system.

**Figure 1:**
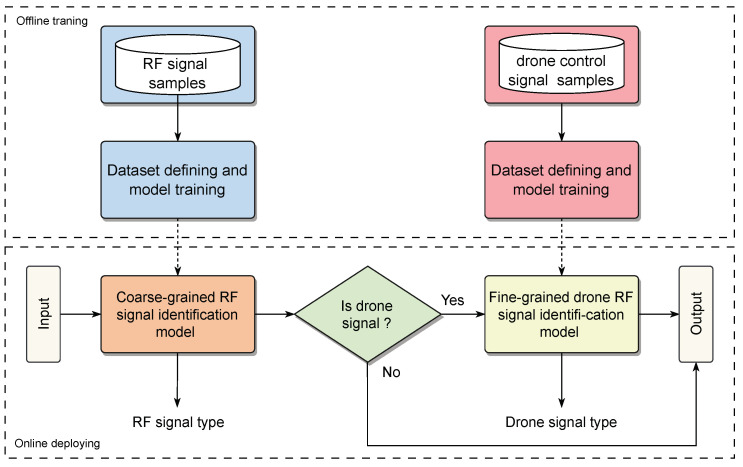
The proposed drone signal RF identification framework.

To address these challenges, we present a comprehensive RF signal identification framework, shown in Figure 1. Our approach encompasses multiple stages that enable the model to ascertain signal origins and drone types with high accuracy. For an *L*-point long drone RF signal waveform $x\in R^{1\times L}$, we first transform the time-domain waveform x to complex T-F spectrum X(m,ω) via the STFT algorithm. This transformation, defined by Equation (1), involves the application of a Hann window w(n) and yields $X\left(m,\omega \right)\in C^{F\times T}$, where *F* and *T* denote the frequency and time dimensions, respectively.

(1) $$X\left(m,\omega \right)=\sum_{n=-\infty }^{+\infty }x\left(n\right)w\left(n-m\right)e^{-j\omega n}$$

**Figure 2:**
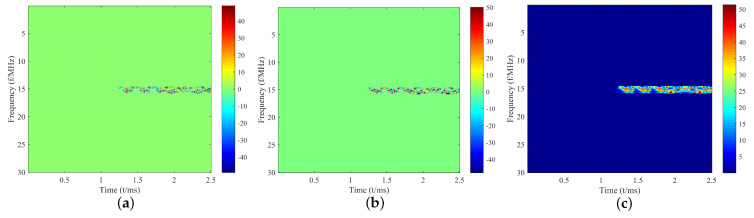
Input data for the proposed model: (**a**) the real part ℜ(X(m,ω)) of the T-F spectrum; (**b**) imaginary part ℑ(X(m,ω)) of the T-F spectrum; (**c**) magnitude part |X(m,ω)| of the T-F spectrum.

While X(m,ω) contains both magnitude |X(m,ω)| and phase information ϕ(X(m,ω)), prior research predominantly focuses on the magnitude component while disregarding phase information. Yet, omitting phase information compromises the completeness of the T-F spectrum, ultimately impacting identification performance. To overcome this limitation, we propose a novel approach: we concatenate the real part ℜ(X(m,ω)), imaginary part ℑ(X(m,ω)), and magnitude part |X(m,ω)| of the T-F spectrum shown in Figure 2. This comprehensive input representation, denoted as $X\in R^{3\times F\times T}$, ensures the model leverages all available signal information, both in magnitude and phase, for more accurate identification.

## 4. Architecture of the Proposed DNN Model

**Figure 3:**
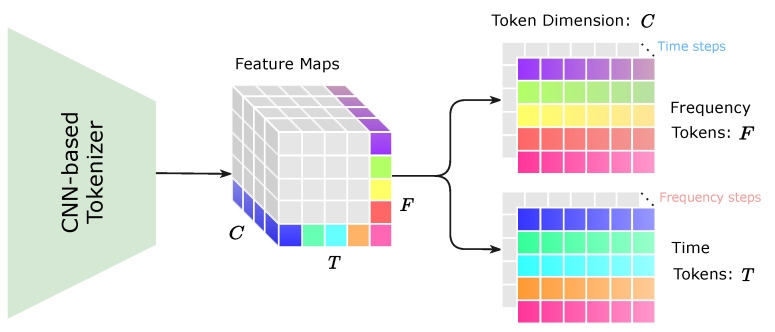
Illustration of the proposed CNN-based tokenization method.

Our chief goal is to design a hybrid transformer model named SignalFormer, which can efficiently capture the local context and global attention map in the drone RF signal T-F spectrum. But the T-F spectrum cannot directly feed into the transformer model because the SA mechanism is acting on a sequence of vectors called tokens. Therefore, the tokenization of the original T-F spectrum is the requisite step for our model. Unlike the non-overlapping patch split and tokenization method of ViT, we propose a CNN-based tokenization method that can generate the tokens with rich local context information for each T-F bin in the feature map (see Figure 3). Then, we design an effective time/frequency transformer approach to capture the global time/frequency correlations between the tokens. For the rest of this section, we first present the pipeline of our SignalFormer architecture. Then, we describe the critical components in SignalFormer: the dilation time–frequency convolution block (D-TFCB), the CNN-based tokenizer (C-tokenizer), the time–frequency downsampling block (TFDB), and the time/frequency transformer encoder (T/F-encoder).

### 4.1. Overall Pipeline

**Figure 4:**
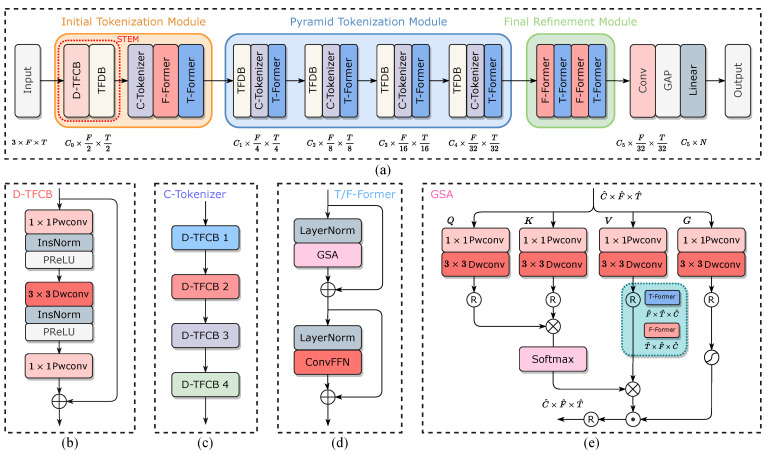
The architecture of the proposed SignalFormer model: (**a**) the overall pipeline of the SignalFormer model; (**b**) the dilation time–frequency convolution block (D-TFCB); (**c**) the CNN-based tokenizer (C-tokenizer); (**d**) the time/frequency transformer encoder (T/F-encoder); and (**e**) the gated self-attention (GSA) mechanism.

Since the drone RF signal identification problem can be formulated as a multi-signal classification task, our SignalFormer model is designed as a classification model that consists of a backbone network for feature extraction and a classifier head for class decisions. Figure 4a shows that the backbone network includes three modules: an initial tokenization module (ITM), a pyramid tokenization module (PTM), and a feature refinement module (FRM). The ITM first deploys a stem part consisting of a D-TFCB and a TFDB block to map the origin input data $X\in R^{3\times F\times T}$ to shadow feature spaces and reduce the time/frequency dimensions to $X_{0}\in R^{C_{0}\times \frac{F}{2}\times \frac{T}{2}}$. Then, the ITM uses a C-tokenizer to generate the tokens to facilitate the following transformer blocks capturing the long-range dependencies of the tokens along the frequency and time axis, respectively. The PTM is a pyramidal network that gradually uses TFDB to extract multi-scale features and increases the token dimensions from $X_{1}\in R^{C_{1}\times \frac{F}{4}\times \frac{T}{4}}$ to $X_{4}\in R^{C_{4}\times \frac{F}{32}\times \frac{T}{32}}$, then uses C-tokenizer and T-encoder to obtain the time token correlations at each frequency bin. The FEM refines feature information from preceding layers through alternating T/F-encoders, subsequently producing well-extracted features for the classifier head. The classifier head contains a 1×1 convolution layer, a global average pooling (GAP) layer, and a linear layer with *N* nodes. The classifier first increases feature channels to $X_{5}\in R^{C_{5}\times \frac{F}{32}\times \frac{T}{32}}$ by the 1×1 convolution layer, and then use the GAP layer to fuse the feature maps. After that, the RF signal class can be decided by the linear layer.

### 4.2. Token Generation Components

To introduce the local context and inductive bias into SignalFormer, we propose several convolutional token generation components (including D-TFCB, C-tokenizer, and TFDB) to replace the non-overlap sliding window generation method in ViT. The following parts will describe these token generation components in detail.

Figure 4b illustrates the structure of D-TFCB. We use two 1×1 point-wise convolution (Pwconv) layers to aggregate pixel-wise cross-channel context and a depth-wise convolution (Dwconv) layer with 3×3 kernel size to capture channel-wise spatial context. Due to the effectiveness of the dilated convolution in time series data processing, we add a dilation factor in the Dwconv layer to obtain a larger receptive field along the time axis. To guarantee the causality of our model, we also use the causal convolution in the Dwconv layer. For D-TFCB, we use the instance normalization (InsNorm) layer and PReLU non-linear function after the first Pwconv layer and the Dwconv layer.

The C-tokenizer is the first core component of our SignalFormer model. Figure 4c shows that the C-tokenizer consists of four stacked D-TFDB blocks with increasing dilation factors. The dilation factors increase exponentially (from 1 to $2^{3}$) to gradually expand the receptive field of the Dwconv layer, obtaining a vast temporal context window at the last D-TFDB block. Assuming the C-tokenizer has *C* output feature channels, then the *C* feature points stacked on the same T-F bin can be considered as a token for this T-F bin (see Figure 3). Finally, we can obtain the T-F tokens for the transformer block.

The TFDB component is used for data downsampling on both the time and frequency axis. Unlike the Dwconv layer in D-TFCB, we use the standard convolution layer with 7×7 kernel size in TFCB to reserve more spatial information. And the kernel stride is (2, 2) in this layer. The TFCB also contains an InsNorm layer and a PReLU non-linear function after the convolution layer.

### 4.3. Time/Frequency Transformer Encoder

The T/F-encoder block is deployed after the C-tokenizer to explore the long-range dependency in the signal spectrum. Figure 4d shows that the T/F-encoder block consists of two-layer normalization (LayerNorm), a gated self-attention (GSA) mechanism, and a convolutional feed-forward network (ConvFFN).

The GSA is the second core component of our SignalFormer model, featuring two operating modes corresponding to the T-encoder and F-encoder. Figure 4e illustrates the detailed structure of the GSA. For a normalized tensor $X_{n}\in R^{\hat{C}\times \hat{F}\times \hat{T}}$, the GSA first projects it to *Query* (Q), *Key* (K), *Value* (V), and *Gate* (G) spaces, this step can be mathematically written as $Q=W_{p}^{Q}W_{d}^{Q}X_{n}$, $K=W_{p}^{K}W_{d}^{K}X_{n}$, $V=W_{p}^{V}W_{d}^{V}X_{n}$, $G=W_{p}^{G}W_{d}^{G}X_{n}$, where $W_{p}^{\left(\cdot \right)}$ and $W_{d}^{\left(\cdot \right)}$ denote the 1×1 Pwconv layer and 3×3 Dwconv layer, respectively. The convolution layers emphasize the local context before accessing the global feature correlations. Next, we can reshape the projections according to the chosen working mode (see the explanation of the reshaping method in Figure 4e): for the T-encoder mode, the dimensions of all projections will be transformed from $\hat{C}\times \hat{F}\times \hat{T}$ to $\hat{F}\times \hat{T}\times \hat{C}$; for the F-encoder mode, the dimension of all projections will be transformed from $\hat{C}\times \hat{F}\times \hat{T}$ to $\hat{T}\times \hat{F}\times \hat{C}$. After that, we can deduce the SA map by the following equation:

(2) $$\mathrm{SA}\left(Q,K,V\right)=\mathrm{Softmax}\left(\frac{QK^{T}}{\sqrt{\hat{C}}}\right)V$$

where $\hat{C}$ is the token dimension. We propose applying a gated mechanism to the SA map to filter out noise components in the tokens, which is beneficial for improving identification performance at low SNR levels. We use the Sigmoid function on the projection G to generate the SA mask, which is then applied to the SA map. The GSA map can be expressed as follows:

(3) GSA(Q,K,V,G)=Sigmoid(G)⊙SA(Q,K,V)

where the operator ⊙ presents the Hadamard product.

The ConvFFN is a sandwich structure network used for integrating feature information from the GSA part. ConvFFN contains two 1×1 Pwconv layers and a 3×3 Dwconv layer. The first Pwconv layer expands the original number of channels by a factor of 4. Next, a Dwconv layer is deployed to encode information from spatially neighboring T-F bins, useful for learning local signal spectrum structure. Then the second Pwconv layer compresses feature channels back to the original input dimension.

### 4.4. Summary of the Model Architecture

In the SignalFormer model, we design four essential components: D-TFCB, C-tokenizer, TFDB, and the T/F-encoder. Then, we use these components to construct the three modules in the proposed model.

For ITM, we use components such as D-TFCB, TFDB, C-tokenizer, and T/F-former. Using ITM can effectively map the original signal time–frequency spectrum to higher dimensional feature spaces, and the application of T/F-former components can complete a global feature extraction before large-scale feature map downsampling, thereby preserving more semantic information in the feature map.

The PTM mainly uses TFDB, C-tokenizer, and T-former components, and we use these components to build a multi-level pyramid network. The PTM module can gradually increase the number of channels in the feature map to obtain higher-level semantic information, while downsampling the feature map to reduce the computational complexity of the model. The use of the T-former component enhances the temporal correlation feature extraction at different frequency bins and filters out the noise components in the T-F spectrum.

The FEM comprises alternating T/F-encoders, which enhance the signal features from the preceding layer; it then ultimately generates well-extracted features for the classifier head.

## 5. Experiment Evaluation

### 5.1. Datasets and Experiment Setup

To validate the performance of the proposed model, two widely used public datasets in the field of drone RF signal identification were applied in this paper: the CARDRF RF Signal [15] and the MPACT drone control signal dataset [16]; these datasets are for coarse-grained and fine-grained signal identification, respectively. Detailed data about the devices contained in the datasets are shown in Table 1 and Table 2.

The CARDRF RF Signal dataset includes four categories of RF signals within the ∼2.4 GHz ISM frequency band: Bluetooth, Wi-Fi, drones, and their respective controllers. Each signal type comprises 2500 samples. On the other hand, the MPACT drone control signal dataset primarily encompasses 15 distinct types of drone control signals, each drone model consisting of 1000 signal samples. Both datasets share a common sampling frequency of 20 GHz, with each sample having a sampling duration of 0.25 ms. The original signal-to-noise ratio (SNR) stands at 30 dB. Specific details regarding the sampling parameters can be found in Table 3.

However, it is challenging in practical engineering to extensively employ expensive high-speed sampling equipment; the high sampling rate of the original signals cannot be satisfied. Furthermore, the high sampling rate results in a significant number of data points within signal samples, posing challenges for the real-time processing of neural networks. Therefore, it requires resampling of the original signal samples at a lower sampling rate. In this paper, we initially convert the signals to baseband and then resample the signals with a 60 MHz sampling frequency, which is easy to fulfill in engineering. In order to investigate the impact of Gaussian white noise and co-channel signal interference on the identification performance of drone control signals, we introduce varying levels of Gaussian white noise and co-channel signal interference to the two datasets, with SNR and signal-to-interference ratio (SIR) ranging from −15 dB to 15 dB. Finally, the coarse-grained and fine-grained signal identification datasets are split into training, validation, and testing sets in an 8:1:1 ratio. Then, we can establish the following experimental schemes on the well-processed datasets to verify the RF signal identification performance of the SignalFormer model under different tasks:

- **Task I:** We use the CARDRF dataset to evaluate the SignalFormer performance for coarse-grained signal identification tasks, including identification tasks under Gaussian white noise (noted as **Task I.a**) and identification tasks under co-channel signal interference (noted as **Task I.b**);
- **Task II:** We use the MPACT dataset to evaluate the identification accuracy of SignalFormer for fine-grained signal identification tasks, including identification tasks under Gaussian white noise (noted as **Task II.a**) and identification tasks under co-channel signal interference (noted as **Task II.b**);

### 5.2. Model Implementation Details

For the implementation details of our SignalFormer, the feature channel numbers in the backbone network $C_{0}$ to $C_{4}$ are {32, 48, 48, 64, and 96}. And the channel number ($C_{5}$) and linear node (*N*) are specific to different tasks: 128 and 4 for task I; 512 and 15 for task II.

**Figure 5:**
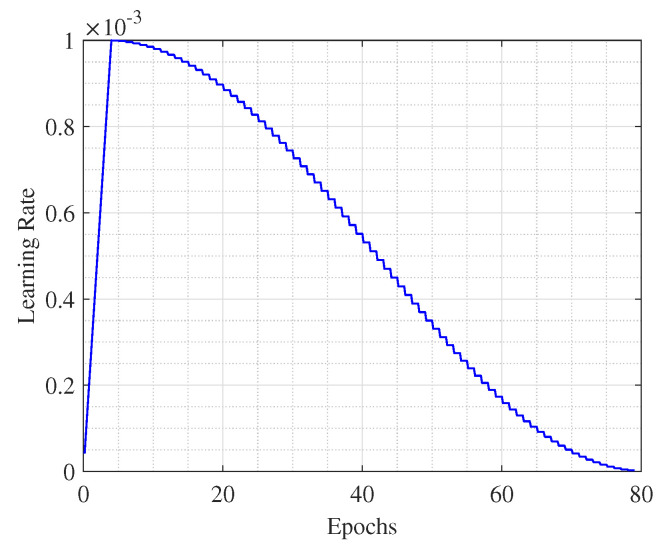
Cosine annealing learning rate curve with a warm-up process.

We train the SignalFormer with the AdamW optimizer (weight decay factor 0.01) for 80 epochs with an initial learning rate of $1\times 10^{-3}$, gradually reducing to $1\times 10^{-6}$ with the cosine annealing, the learning curve is shown in Figure 5. Before the regular training, we warm up the SignalFormer model for three epochs. The batch size is 48. The detailed hyperparameters are listed in Table 4. For the loss function in the training process, we choose the cross-entropy function, as follows:

(4) $$L=-\frac{1}{N}\sum_{i=1}^{N}\sum_{c=1}^{M}y_{ic}\log\left(p_{ic}\right)$$

where *N* is the batch size, *M* represents the total class number, $y_{ic}$ presents the binary indicator (equal to 1 if class label *c* is the correct classification for sample *i*, otherwise, it is equal to 0), $p_{ic}$ represents the predicted probability that *i* belongs to class label *c*.

### 5.3. Baseline Models

Within this paper, we select a range of contemporary deep neural network (DNN) models as baseline benchmarks. These encompass CNN-based architectures, like ResNet [26], RegNet [27], EfficientNet [28], as well as the transformer-based model ViT [29]. To mitigate overfitting concerns, we choose the most compact model configurations from their respective model repositories.

## 6. Experimental Results

### 6.1. Comparative Experiments on Coarse-Grained Signal Identification Tasks

Table 5 shows detailed experimental results for the coarse-grained signal identification task. From the perspective of the spatial complexity of the model, the parameter size of the SignalFormer model is only 1.66 million, which occupies a smaller memory space in the actual deployment. However, due to the extensive use of convolution and SA mechanisms in the SignalFormer model, the computational complexity of the SignalFormer model reached 2.99 GFLOPs, which is higher than other models. From the perspective of the coarse-grained signal identification accuracy of the model, the SignalFormer model can achieve high identification accuracy in environments such as Gaussian white noise or co-frequency signal interference: the average accuracy under −15 dB∼15 dB Gaussian white noise is 97.57%, outperforming the second RegNet model by 1.43% and the Waveformer model by 0.73%; the accuracy in the interference environment of −15 dB∼15 dB co-frequency signal is 98.03%, outperforming the second RegNet model by 1.15% and the Waveformer model by 1.38%. The above experimental results show that the SignalFormer model proposed in this paper can effectively achieve coarse-grained identification of drone control signals and occupy less space. However, by using both convolution and self-attention feature extraction mechanisms in the SignalFormer model, the accuracy of signal identification is improved, but the computational delay of the model is increased.

**Figure 6:**
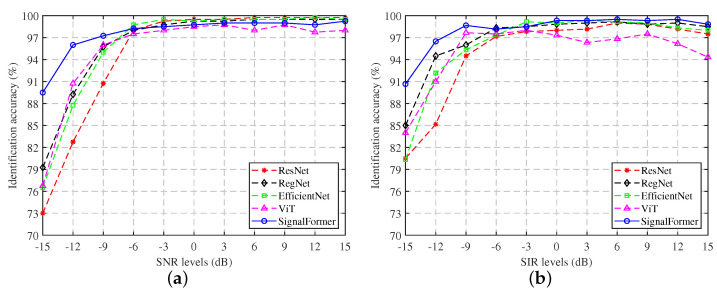
Coarse-grained signal identification accuracy under different SNR/SIR conditions: (**a**) Gaussian white noise environment; (**b**) co-frequency signal interference environment.

Figure 6a shows the coarse-grained signal identification performance of several models under different SNR conditions in a Gaussian white noise environment. It can be seen that the SignalFormer model can significantly outperform other models in accuracy under low SNRs of −15 dB to −6 dB. When the SNR is −15 dB, the accuracy of SignalFormer is 89.5%, surpassing the RegNet model by 10.25% and surpassing the ViT model (which also uses the self-attention mechanism) by 12.75%. When the SNR is higher than −6 dB, the accuracy of the SignalFormer model will be slightly lower than other convolutional neural network models, but the accuracy can still be maintained at around 99%, meeting the needs of control signal identification tasks. Figure 6b shows the performance comparison of several models under different SIR conditions in the co-frequency interference environment. It can be seen that the impact of the co-frequency signal interference on identification accuracy is weaker than that of Gaussian white noise. In the low SIR range of −15 dB∼−6 dB, the identification accuracy of the five models is improved compared to the Gaussian white noise environment. When the SIR is −15 dB, the accuracy of the SignalFormer model is close to 91%, which is nearly 6% higher than the RegNet model. When the SIR is higher than −6 dB, the SignalFormer model can still maintain the highest accuracy, only occasionally falling below the EfficientNet model at −3 dB.

### 6.2. Comparative Experiments on Fine-Grained Signal Identification Task

In the drone RF signal identification task, in addition to solving the problem of coarse-grained identification of the drone control signal with other co-frequency RF signals, it is also necessary to solve the problem of fine-grained identification of different drone control signal categories, so this subsection focuses on the study of comparing the fine-grained signal identification performance of different models. The experimental results are shown in Table 6, from which it can be seen that the average identification accuracy of the SignalFormer model in the Gaussian white noise environment of −15 dB∼15 dB is 97.48%, which is higher than that of the second EfficientNet model by 0.71%. The average identification accuracy of the SignalFormer model in the co-frequency interference environment is 98.16%, which is higher than that of the RegNet model at 1.60%. The above experimental results show that the SignalFormer model cannot only solve the coarse-grained identification problem of RF signals but also effectively deal with the fine-grained signal identification problem of drone control signals.

**Figure 7:**
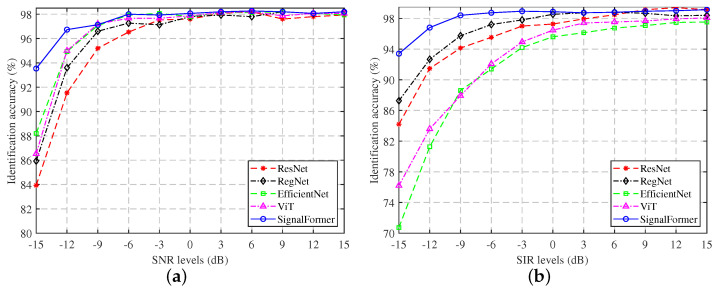
Fine-grained signal identification accuracy under different SNR/SIR conditions: (**a**) Gaussian white noise environment; (**b**) co-frequency signal interference environment.

To explore the impact of Gaussian white noise and co-frequency signal interference on the fine-grained signal identification performance of the models, we conducted tests to assess the identification accuracy of five neural network models under various conditions. Figure 7a illustrates the fine-grained signal identification accuracy of different models in a Gaussian white noise environment ranging from −15 dB to 15 dB. Notably, the SignalFormer model demonstrates exceptional identification performance in the SNR range of −15 dB to −9 dB, significantly surpassing other models. As the SNR exceeds −9 dB, the SignalFormer model maintains an accuracy level of approximately 98%, placing it on par with convolutional models like EfficientNet.

Figure 7b presents the model’s identification performance in a co-frequency interference environment. It is evident that co-frequency interference profoundly affects the performance of the EfficientNet and ViT models. When the SIR falls below −9 dB, their accuracy drops significantly below 90%, making them unsuitable for practical applications. In contrast, the SignalFormer model experiences a relatively minor impact from co-frequency interference. This resilience stems from SignalFormer’s capacity to effectively distinguish drone control signal features from co-frequency interference through its attention mechanism. Additionally, the convolution mechanism ensures the model’s ability to extract small local features efficiently.

**Figure 8:**
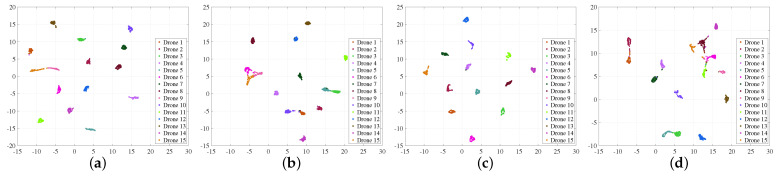
Visualization of feature clustering for signal identification: (**a**) Gaussian white noise environment (SNR = 15 dB); (**b**) Gaussian white noise environment (SNR = −15 dB); (**c**) co-frequency interference environment (SIR = 15 dB); (**d**) co-frequency interference environment (SIR = −15 dB).

To gain an intuitive understanding of the SignalFormer model’s feature extraction capabilities, we applied the unified manifold approximation and projection (UMAP) algorithm [30] to reduce the dimensionality of the feature vectors extracted from the SignalFormer model. Subsequently, we conducted clustering on these diverse features, and the clustering outcomes are visualized in Figure 8. This figure reveals that when SNR/SIR is high, the feature clusters for various categories of drone control signals remain distinctly separate with no overlap. However, at an SNR/SIR of −15 dB, we observe an aliasing phenomenon in the clustering of certain signal samples. This phenomenon indicates that the feature data of the signal become difficult to differentiate due to Gaussian white noise or co-frequency interference, resulting in a decrease in identification accuracy.

### 6.3. Ablation Studies

**Figure 9:**
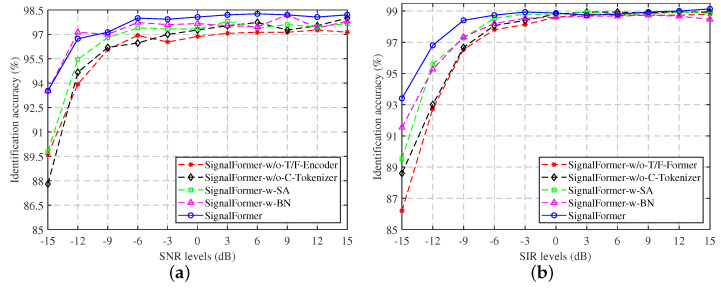
The ablation studies results under different SNR/SIR conditions: (**a**) Gaussian white noise environment; (**b**) co-frequency signal interference environment.

The ablation study was conducted on the task II datasets to investigate the influence of various components on signal identification performance. The results of the explicit experiments are presented in Table 7 and Figure 9. In our initial investigation, we focused on the model’s performance after the removal of the T/F-encoder component, which includes the self-attention mechanism (referred to as SignalFormer-w/o-T/F-encoder). As shown in Table 7, it is evident that although this modification resulted in a reduction of 0.74 M parameters and 0.88 GFLOPs in computational cost, the identification accuracy drop was 1.58% and 1.45% under Gaussian white noise and co-frequency signal interference conditions, respectively. These findings indicate that despite the self-attention mechanism potentially increasing model complexity, it remains effective in extracting features from the signal’s time spectrum, thereby improving overall model performance.

Next, we examined the model’s identification performance when utilizing token vector encoding without the inclusion of the C-tokenizer component (referred to as SignalFormer-w/o-C-tokenizer). In this configuration, the model’s accuracy decreased by 1.35% and 1.06% under Gaussian white noise and co-frequency signal interference conditions, respectively. However, it is worth noting that even with the exclusion of the C-tokenizer component, which predominantly employs deep separable convolutions, the model’s complexity did not experience a significant reduction. These experimental findings underscore the effectiveness of the C-tokenizer component in extracting local features from the time–frequency spectrum and facilitating Token vector encoding for robust control signal identification.

Then, we experimented by replacing the gated self-attention mechanism with a regular self-attention mechanism (referred to as SignalFormer-w-SA) to assess the effectiveness of the gated mechanism in signal identification tasks. The results show that the gated mechanism enhances the model’s accuracy under Gaussian white noise and co-frequency signal interference conditions by 0.93% and 0.63%, respectively. This improvement comes with only a minimal increase of 0.06M in parameter quantity and 0.08 GFLOPs in computational cost. From Figure 9, it is evident that the performance enhancement due to the gating attention mechanism primarily occurs in low SNR conditions. These findings suggest that the gated mechanism effectively filters out noise components from the features.

Lastly, we replaced the LN layer with a batch normalization (BN) layer (referred to as SignalFormer-w-BN), which led to a slight decrease in the model’s accuracy under Gaussian white noise and co-frequency signal interference conditions, by 0.29% and 0.64%, respectively. This change occurred because the BN layer scales each feature channel of all samples within a batch, while the LN layer scales all feature channels of each sample. However, since each drone control signal may have different energy levels, using a BN layer can distort the extracted signal features.

Additionally, to assess the impact of input data types on the identification model’s performance, we evaluated the five models with three types of input data: the magnitude of the spectrum |X(m,ω)|, the complex spectrum X(m,ω), and the concatenated magnitude and complex spectrum X. As presented in Table 8 and Table 9, it is evident that employing the magnitude spectrum concatenated with the complex spectrum as input data effectively enhances the identification performance for all models, irrespective of the presence of Gaussian white noise or co-frequency signal interference. These experimental results underscore the significance of both phase and magnitude information in the T-F spectrum for drone signal identification.

### 6.4. Class-Incremental Learning Studies

While the above experiments have showcased the robust identification performance of the SignalFormer model, it remains essential to assess the model’s class-incremental learning (CIL) capabilities for handling the potential emergence of new drone control signal categories—post-deployment—from an engineering practicality perspective. Subsequent experiments are conducted to compare the incremental learning abilities of the SignalFormer model with other models.

**Figure 10:**
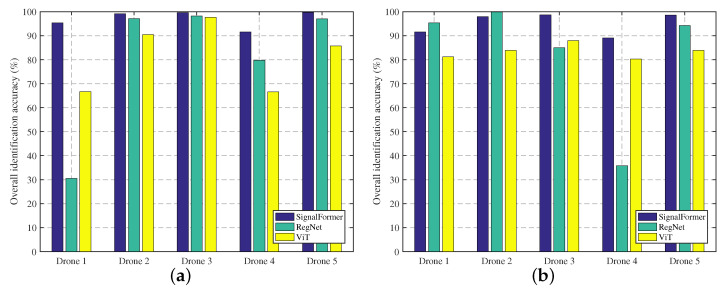
Comparison of incremental learning abilities of different models: (**a**) Gaussian white noise environment; (**b**) co-frequency signal interference environment.

Initially, a foundational dataset consisting of 10 categories of drone control signals that are randomly selected from the fine-grained identification dataset of drones is used to train three models: SignalFormer, RegNet, and ViT. Then, the remaining five signal categories are employed to fine-tune these models and evaluate their accuracy in detecting signals from those categories. Figure 10a displays the average identification accuracy of the three models for the control signals of five new drone categories under −15 dB∼15 dB Gaussian white noise. The results demonstrate that the SignalFormer model consistently outperforms the other models in CIL performance, maintaining an identification accuracy exceeding 90%. In contrast, the RegNet and ViT models may exhibit lower accuracy for specific drone categories, indicating weaker model stability. Figure 10b illustrates the CIL performance of the three models in an environment with the same frequency signal interference. The experimental outcomes in the figure further emphasize SignalFormer’s robust incremental learning ability, with over 90% identification accuracy and only slightly below 90% on drone type 4.

### 6.5. Results Discussion

In the first experiment, we demonstrate that the proposed method outperforms other methods on the coarse-grained signal identification task. The proposed SignalFormer reaches 97.57% with Gaussian noise and 98.03% in the presence of co-frequency interference. In the second experiment, we demonstrate that the proposed method outperforms other methods on the fine-grained signal identification task with, respectively, 97.48% under Gaussian noise conditions and 98.16% in the presence of co-frequency interference. These two experiments show the advantage of the proposed method in performing ADI tasks in different scenarios. In the third experiment, the contributions of different components of the proposed SignalFormer were evaluated. The results show the efficiency of the gated self-attention mechanism in capturing global time/frequency correlations among these T-F tokens and underscore the importance of phase information signal identification. These findings have implications for other spectrum-based classification and recognition tasks. In the last experiment, the incremental learning ability of the proposed method is evaluated. The well-trained SignalFormer model can easily achieve good performance on different ADI tasks with minimal fine-tuning, demonstrating the strong generalization ability of the proposed SignalFormer.

Briefly, the proposed method has achieved better identification performance on the two open datasets, CARDRF and MPACT. Even though the number of drone types is only a small part of the number of drone types already in existence, as SignalFormer shows strong generalization ability in the experiment of incremental learning abilities, we believe that SignalFormer is competent for various ADI tasks. Since the features of RF signals depend strongly on the RF chips and the algorithms implemented on the drones, an RF-based ADI method cannot identify two different drone models that come from the same manufacturer and use the same RF chip. This fact suggests a fusion of various ADI methods in the future to benefit from the advantages of each method and refine the identification ability to perform ADI tasks in various scenarios. Moreover, since SignalFormer uses features in the frequency spectrum domain, the processing speed is limited. Therefore, in order to deploy SignalFormer in practical applications, the processing speed also needs to be improved in the future.

## 7. Conclusions

In this paper, we propose a hybrid transformer model designed to effectively identify drone RF signals within the aerial RF environment. Our approach encompasses several key innovations. Firstly, we developed a CNN-based tokenization method capable of generating T-F tokens enriched with substantial local context information for each T-F bin. We coupled this with an efficient gated self-attention mechanism to capture global time/frequency correlations among these T-F tokens. Subsequently, we constructed and trained the SignalFormer model to perform both coarse-grained RF signal identification and fine-grained drone control signal identification. Moreover, we highlighted the significant impact of incorporating phase information into the input of the SignalFormer model, revealing its vital role in enhancing model performance. This underscores the critical importance of phase information within the T-F spectrum for drone RF signal identification, which has implications for other spectrum-based classification and recognition tasks.

In the experimental stage, we conducted a series of rigorous tests under Gaussian white noise and co-frequency signal interference conditions to assess SignalFormer’s capabilities and robustness. We also introduced a class-incremental learning evaluation to showcase SignalFormer’s competence in handling previously unseen categories of drone signals. The results of these experiments consistently demonstrated the high identification accuracy of our proposed model in terms of drone RF signal identification. Looking ahead, we anticipate further refinements to SignalFormer, including exploring its application in real-world scenarios and investigating its potential for adapting to dynamic signal environments. Additionally, we plan to continue extending its capabilities to address emerging challenges in drone RF signal identification.

## Figures and Tables

**Table 1:** Catalog of RF devices in the CARDRF dataset.

Device	Maker	Model
UAV	DJI	Phantom 4
	DJI	Inspire
	DJI	Matrice 600
	DJI	Mavic Pro 1
	BeebeeRun	FPV RC drone mini quadcopter
	3DR	Iris FS-TH9x
Bluetooth	Apple	iPhone 6S
	Apple	iPhone 7
	Apple	iPad 3
	Fitbit	Charge3 smartwatch
	Motorola	Motorola E
Wi-Fi	Cisco	Linksys E3200
	TP-Link	TL-WR940N

**Table 2:** Drone catalog in the MPACT dataset.

Maker	Model	Maker	Model
DJI	Inspire 1 Pro	Spektrum	DX5e
DJI	Matrice 100	Spektrum	DX6e
DJI	Matrice 600	Spektrum	DX6i
DJI	Phantom 4 Pro	Spektrum	JR X9303
DJI	Phantom 3	Graupner	MC-32
Futaba	T8FG	FlySky	FS-T6
HobbyKing	HK-T6A	Jeti Duplex	DC-16
Turnigy	9X	-	-

**Table 3:** The sampling parameters of the CARDRF and MPACT datasets.

Sampling Parameters	Values
Sampling frequency	20 GSA/s
Carrier frequency	∼2.4 GHz
Time duration	0.25 ms
SNR level	30 dB

**Table 4:** Hyperparameter setting.

Hyperparameters	Values
Optimizer	AdamW
Base learning rate	$1\times 10^{-3}$
Final learning rate	$1\times 10^{-6}$
Weight decay	0.01
Optimizer momentum	(0.9, 0.999)
Scheduler	Cosine annealing
Batch size	48
Warm-up epochs	3
Training epochs	80
Drop path	0.01

**Table 5:** Comparison of experimental results for coarse-grained signal identification performance.

Method	#Params	FLOPs	Task I.a	Task I.b
ResNet	4.93 M	1.80 G	94.70%	94.98%
RegNet	2.32 M	0.32 G	96.14%	96.88%
EfficientNet	4.03 M	0.63 G	95.86%	96.11%
ViT	10.34 M	1.66 G	95.34%	95.15%
SignalFormer	1.66 M	2.99 G	**97.57%**	**98.03%**

**Table 6:** Comparison of experimental results for the fine-grained signal identification performance.

Method	#Params	FLOPs	Task II.a	Task I.b
ResNet	4.93 M	1.80 G	95.63%	95.79%
RegNet	2.32 M	0.32 G	96.23%	96.56%
EfficientNet	4.03 M	0.63 G	96.77%	91.52%
ViT	10.34 M	1.66 G	96.57%	92.71%
SignalFormer	1.66 M	2.99 G	**97.48%**	**98.16%**

**Table 7:** The experimental results of ablation studies.

Method	#Params	FLOPs	Task II.a	Task II.b
SignalFormer-w/o-T/F-encoder	0.92 M	2.11 G	95.90%	96.71%
SignalFormer-w/o-C-tokenizer	1.49 M	2.64 G	96.13%	97.10%
SignalFormer-w-SA	1.60 M	2.91 G	96.55%	97.53%
SignalFormer-w-BN	1.66 M	2.99 G	97.19%	97.52%
SignalFormer	1.66 M	2.99 G	**97.48%**	**98.16%**

**Table 8:** Influence of input data type on identification performance in a Gaussian white noise environment.

Method	Input Type	−15 dB	−12 dB	−9 dB	−6 dB	−3 dB	0 dB	3 dB	6 dB	9 dB	12 dB	15 dB	Average
ResNet	Magnitude	77.40%	88.27%	92.73%	94.67%	95.00%	95.87%	96.00%	95.87%	97.20%	96.93%	97.33%	93.39%
	Complex	73.73%	89.87%	94.47%	96.40%	97.00%	97.53%	97.87%	97.80%	**97.93%**	**97.93%**	97.87%	94.40%
	Combined	**83.93%**	**91.53%**	**95.20%**	**96.53%**	**97.53%**	**97.60%**	**98.07%**	**98.20%**	97.60%	97.80%	**97.93%**	**95.63%**
RegNet	Magnitude	81.07%	89.67%	92.53%	94.60%	94.27%	95.40%	95.33%	95.80%	96.00%	95.93%	96.47%	93.37%
	Complex	**86.60%**	**93.73%**	96.47%	96.40%	96.53%	97.33%	96.40%	96.33%	96.40%	95.93%	96.07%	95.29%
	Combined	85.93%	93.60%	**96.60%**	**97.27%**	**97.13%**	**97.80%**	**97.93%**	**97.80%**	**98.20%**	**98.07%**	**98.20%**	**96.23%**
Efficient- Net	Magnitude	78.27%	86.27%	91.73%	94.47%	95.53%	96.73%	97.00%	97.73%	98.27%	98.40%	98.67%	93.92%
	Complex	80.07%	91.13%	95.60%	96.93%	98.00%	**98.00%**	**98.27%**	**98.47%**	**98.60%**	**98.73%**	**98.80%**	95.69%
	Combined	**88.20%**	**94.93%**	**97.07%**	**97.93%**	**98.07%**	97.80%	98.13%	98.13%	98.20%	98.07%	97.93%	**96.77%**
ViT	Magnitude	85.27%	91.60%	92.73%	94.67%	94.73%	96.00%	95.40%	95.73%	95.67%	95.87%	96.00%	93.97%
	Complex	68.87%	85.60%	92.87%	94.27%	95.60%	96.20%	96.13%	96.20%	96.53%	96.87%	96.20%	92.30%
	Combined	**86.53%**	**95.00%**	**97.20%**	**97.67%**	**97.67%**	**97.87%**	**98.13%**	**98.20%**	**97.87%**	**98.07%**	**98.07%**	**96.57%**
Signal- Former	Magnitude	83.20%	90.47%	92.07%	92.67%	93.87%	95.07%	96.07%	96.27%	96.20%	96.60%	96.47%	93.54%
	Complex	90.73%	95.73%	**97.27%**	97.40%	97.33%	97.73%	98.00%	97.87%	97.93%	98.07%	97.73%	96.89%
	Combined	**93.53%**	**96.73%**	97.13%	**98.00%**	**97.93%**	**98.07%**	**98.20%**	**98.27%**	**98.20%**	**98.07%**	**98.20%**	**97.48%**

**Table 9:** Influence of input data type on identification performance in the co-frequency environment.

Method	Input Type	−15 dB	−12 dB	−9 dB	−6 dB	−3 dB	0 dB	3 dB	6 dB	9 dB	12 dB	15 dB	Average
ResNet	Magnitude	83.33%	90.53%	92.80%	94.67%	94.93%	96.53%	97.00%	97.33%	97.60%	97.33%	97.27%	94.48%
	Complex	80.00%	88.53%	93.00%	**95.60%**	**97.60%**	**98.47%**	**98.87%**	**98.87%**	**98.87%**	98.87%	99.00%	95.24%
	Combined	**84.20%**	**91.47%**	**94.13%**	95.53%	97.00%	97.27%	97.93%	98.47%	**99.13%**	**99.40%**	**99.13%**	**95.79%**
RegNet	Magnitude	87.13%	90.67%	93.33%	96.33%	97.67%	98.53%	98.67%	98.47%	98.00%	97.87%	98.20%	95.90%
	Complex	84.40%	91.13%	94.07%	96.80%	**98.00%**	**98.67%**	**98.87%**	**98.87%**	**98.73%**	**98.80%**	**98.93%**	96.12%
	Combined	**87.27%**	**92.67%**	**95.73%**	**97.20%**	97.80%	98.53%	98.73%	98.80%	98.67%	98.33%	98.40%	**96.56%**
Efficient- Net	Magnitude	**77.27%**	**84.67%**	86.73%	89.33%	92.20%	94.00%	94.67%	95.00%	95.00%	95.27%	95.07%	90.84%
	Complex	66.00%	78.60%	85.67%	90.93%	94.00%	**95.93%**	**96.13%**	**97.33%**	**97.73%**	**97.80%**	**97.87%**	90.73%
	Combined	70.73%	81.27%	**88.60%**	**91.40%**	**94.20%**	95.60%	**96.13%**	96.73%	97.07%	97.47%	97.53%	**91.52%**
ViT	Magnitude	65.73%	69.80%	74.27%	77.33%	84.47%	90.20%	91.67%	92.93%	93.80%	94.07%	94.27%	84.41%
	Complex	56.67%	69.20%	76.33%	82.87%	88.53%	92.60%	94.33%	95.00%	95.60%	95.80%	96.07%	85.73%
	Combined	**76.20%**	**83.60%**	**87.93%**	**92.07%**	**94.93%**	**96.47%**	**97.40%**	**97.53%**	**97.67%**	**97.93%**	**98.07%**	**92.71%**
Signal- Former	Magnitude	89.87%	93.87%	96.47%	98.13%	98.27%	98.47%	**98.73%**	99.07%	**99.13%**	**99.00%**	98.93%	97.27%
	Complex	90.87%	95.87%	97.80%	98.13%	98.47%	98.60%	**98.73%**	98.73%	98.73%	98.53%	98.53%	97.55%
	Combined	**93.40%**	**96.80%**	**98.40%**	**98.73%**	**98.93%**	**98.87%**	**98.73%**	**98.80%**	98.93%	**99.00%**	**99.13%**	**98.16%**

## Data Availability

Two publicly available datasets, namely the Cardinal Radio Frequency (CARDRF) dataset and the MPACT Lab Drone Remote Controller RF Signal (MPACT) dataset, were used to illustrate and evaluate the proposed architecture.
